# Healthy eating: a matter of prioritisation by households or policymakers?

**DOI:** 10.1017/S1368980021000677

**Published:** 2021-05

**Authors:** Joreintje Dingena Mackenbach

**Affiliations:** 1 Department of Epidemiology and Data Science, Amsterdam UMC–Vrije Universiteit, De Boelelaan 1089a, 1081HV Amsterdam, The Netherlands; 2 Upstream Team, Amsterdam UMC, Amsterdam, The Netherlands Email: j.mackenbach@amsterdamumc.nl

## Abstract

I reflect upon the potential reasons why American low-income households do not spend an optimal proportion of their food budgets on fruits and vegetables, even though this would allow them to meet the recommended levels of fruit and vegetable consumption. Other priorities than health, automatic decision-making processes and access to healthy foods play a role, but solutions for the persistent socio-economic inequalities in diet should be sought in the wider food system which promotes cheap, mass-produced foods. I argue that, ultimately, healthy eating is not a matter of prioritisation by individual households but by policymakers.

Low fruit and vegetable (F&V) intake is a leading dietary risk factor for morbidity and mortality from non-communicable diseases (NCD)^(1)^. Yet, consumption of F&V remains below the recommended levels in most countries^(2,3)^. Moreover, F&V consumption is socio-economically patterned, with individuals with lower education and income levels having lower levels of F&V consumption than those with higher socio-economic position^(4,5)^. One of the barriers to consuming sufficient levels of F&V is the (perceived) cost of healthy food^(6–13)^. The US Supplemental Nutrition Assistance Program (SNAP) addresses this barrier by providing nutrition benefits to supplement the food budget of families at or below 130 % of the poverty line^(14)^.

In their manuscript ‘The More Households Prioritize Healthy Eating, The Better They Can Afford to Consume a Sufficient Quantity and Variety of Fruits and Vegetables’, Stewart *et al.* suggest that low-income households that prioritise healthy eating by allocating around 40 % of their SNAP benefits to F&V can consume a reasonable variety of F&V each week. However, actual proportion spending on F&V by American families is closer to 25 %^(15)^, which would make adhering to F&V guidelines only feasible by exclusively selecting the cheapest but potentially less palatable F&V. Of course, there are many explanations for why low-income families do not spend 40 % of their SNAP benefits on F&V, and the authors mention factors such as time constraints, lack of cooking skills, food preferences and lack of budgeting skills. Yet, even with additional education around budgeting, shopping and cooking skills, as also provided by SNAP-Ed^(16)^, SNAP recipients have a persistent poor diet score^(17)^.

Dual process theories^(18,19)^ offer an explanation for why individuals do not make ‘optimal’ choices given their budgetary constraints: food choices are not only the result of slow, deliberate thinking processes in which different options are carefully weighted, but also the result of a faster, reactive and intuitive process. Especially under financial and other types of stress, food choices are more likely to be automatic and less reason-based. And even if food choices are made rationally, reaching satiety, preventing food waste and taste preferences of household members may be considered more important than health considerations.

Stewart *et al.* also refer to the fact that some drivers of food choices are out of the control of the household: indeed, households are dependent on having access to lower-priced supermarkets in order to purchase F&V for prices that match their budgets^(20–22)^. This recognition of the wider upstream^(23)^ and systemic^(24)^ determinants of food choices is crucial in understanding and addressing the challenge of low F&V consumption. Indeed, for individuals living in ‘obesogenic’ food environments, where unhealthy foods are available everywhere and heavily marketed, automatic food choices are likely to be unhealthy.

Still, many policy responses, including SNAP-Ed, are highly ‘agentic’, i.e. they require individuals to use their personal resources to benefit from the intervention, even though these approaches have demonstrated low effectiveness^(25)^. Changing the environments in which people make food choices (e.g. through ‘choice architecture’^(26)^ or ‘nudging’^(27)^) has therefore been proposed as a promising strategy to make healthier food choices easier and is gaining traction among researchers^(28)^ and policymakers^(29)^. The popularity of nudging among policymakers is attributable to its liberty-preserving approach that rules out significant financial incentives or regulation to change individual behaviours^(29)^.

However, policymakers are not the only actors trying to influence the food choice architecture; large food corporations may use ‘dark nudges’ to trick consumers into making food choices that are against their best interests^(30)^. Similarly, the term ‘sludge’ refers to the practice of using individuals’ cognitive biases to make health-promoting behavioural changes harder^(30)^. This is reflected by the fact that half of the calories consumed by Americans come from ultra-processed foods^(31)^ despite its associated health risks^(32)^. These challenges highlight the fundamental misalignment between public health goals and the wider food system^(33)^, and using nudges to get people to eat healthier may be regarded as a superficial repair of a food system that promotes the consumption of cheap, appealing, ultra-processed and energy-dense products^(24,34,35)^.

To truly address persistent poor dietary intake and its health consequences, a significant shift in thinking, focused on transforming the food system rather than patchwork solutions, is required. By providing SNAP benefits, the government is essentially competing with the artificially low prices of unhealthy foods that do not reflect the external costs to society such as obesity and greenhouse gas emissions^(36)^. Without governmental regulations, it is likely that a complex adaptive system such as the food system will maintain an equilibrium that benefits large food companies rather than public health^(35,37)^. Shifting this equilibrium in such a way that it provides a solid basis for healthier food choices and creates new and sustainable business models for food industry actors will likely take fiscal policies such as taxes on sugary drinks and junk food, regulation of unhealthy food marketing, mandating front-of-pack food labelling and reducing commercial influences on food policies^(24,30,35)^. Of course, what this requires is bold prioritisation by policymakers, rather than by individual households.
